# Self-Attention Mechanism-Based Head Pose Estimation Network with Fusion of Point Cloud and Image Features

**DOI:** 10.3390/s23249894

**Published:** 2023-12-18

**Authors:** Kui Chen, Zhaofu Wu, Jianwei Huang, Yiming Su

**Affiliations:** 1 College of Civil Engineering, Hefei University of Technology, Hefei 230009, China; 2021170777@mail.hfut.edu.cn (K.C.); hjw1028@hfut.edu.cn (J.H.); 2022170664@mail.hfut.edu.cn (Y.S.); 2 Key Laboratory of Geospatial Technology for the Middle and Lower Yellow River Regions, Henan University, Ministry of Education, Kaifeng 475004, China

**Keywords:** point cloud data, head pose estimation, PointNet, ResNet, multi-source feature fusion

## Abstract

Head pose estimation serves various applications, such as gaze estimation, fatigue-driven detection, and virtual reality. Nonetheless, achieving precise and efficient predictions remains challenging owing to the reliance on singular data sources. Therefore, this study introduces a technique involving multimodal feature fusion to elevate head pose estimation accuracy. The proposed method amalgamates data derived from diverse sources, including RGB and depth images, to construct a comprehensive three-dimensional representation of the head, commonly referred to as a point cloud. The noteworthy innovations of this method encompass a residual multilayer perceptron structure within PointNet, designed to tackle gradient-related challenges, along with spatial self-attention mechanisms aimed at noise reduction. The enhanced PointNet and ResNet networks are utilized to extract features from both point clouds and images. These extracted features undergo fusion. Furthermore, the incorporation of a scoring module strengthens robustness, particularly in scenarios involving facial occlusion. This is achieved by preserving features from the highest-scoring point cloud. Additionally, a prediction module is employed, combining classification and regression methodologies to accurately estimate head poses. The proposed method improves the accuracy and robustness of head pose estimation, especially in cases involving facial obstructions. These advancements are substantiated by experiments conducted using the BIWI dataset, demonstrating the superiority of this method over existing techniques.

## 1. Introduction

The accurate determination of head poses forms the foundation of human–computer interaction and facial analysis. Head pose estimation is widely applicable across numerous fields, such as line-of-sight estimation [1], human–computer interaction [2,3,4,5], and recognition of driving attention [6].

The estimation of head pose recognition can be divided into two categories based on the method used: head pose estimation with facial features and head pose estimation without facial features. Head pose estimation with feature points relies on determining a head’s orientation through the relative positions of facial feature points on the human face. In [7], researchers utilized OpenFace(2.2.0), an open-source software, to extract facial feature points and applied the Solve-Perspective-n-Point (SolvePnP) algorithm for head pose estimation. Experiments conducted on the BIWI dataset achieved absolute errors of 7.9°, 5.6°, and 4.5° for the yaw, roll, and pitch angles, respectively. However, the estimation of a head pose from 2D feature estimation yields lower accuracy owing to the necessity of 3D information. In [8], researchers employed a cascade regression tree model to localize facial feature points on the human body using a stereo camera setup to recover these feature points’ three-dimensional (3D) coordinates. Utilizing the 3D information of the feature points led to more accurate head pose estimations, particularly in the range from −15° to 15° of head rotation. Consequently, utilizing 3D information enhances the accuracy of head pose estimation compared to relying solely on two-dimensional (2D) feature points. While the use of binocular cameras has shown favorable results in small-scale head pose estimations, its accuracy diminishes considerably when applied to large-scale head pose estimations. Zhao et al. [9] proposed a head pose tracking method based on scale-invariant feature transform (SIFT) feature point matching. This method detects SIFT feature points in two consecutive frames and estimates the head pose by calculating a rotation matrix based on the 3D information of the matched feature points. This approach addresses the challenge of reduced estimation accuracy under large pose angles, which stems from imprecisions in feature point localization. However, error accumulation in estimating continuous head poses remains a concern. Liu et al. [10] reconstructed a 3D face model using facial key-point coordinates for head pose estimation, achieving an average accuracy of 7.05° on the BIWI dataset. Head pose estimation without feature points relies primarily on machine learning and deep learning methods [11,12,13,14], which demonstrate flexibility and robustness in handling occlusions and extreme pose variations. Ruiz et al. [15] introduced a novel network named HopeNet, which accepts RGB images as input, utilizes ResNet50 to extract image features, and transforms the regression problem into a classification problem, thereby addressing head pose estimation without landmarks. This convolutional neural network (CNN)-based head pose estimation approach demonstrated higher accuracy and stability than methods relying on feature points. However, this method’s dependency on ResNet50 as the backbone compromises its real-time applicability. Inspired by the soft stagewise regression network (SSRNet), Yang et al. [16] introduced the fine-grained structure aggregation network (FSANet), which surpassed the HopeNet network in terms of both speed and accuracy. While training the HopeNet network, definite losses computation using one-hot labels introduces significant errors when classifying two samples with slight differences. Advancements in deep learning have led to the preprocessing of data labels to enable models to capture high-frequency information from data. Label distribution learning was demonstrated to be effective in [17,18], employing soft labels generated using Gaussian functions instead of one-hot vectors for training head pose models [19]. These methods demonstrated improvements over the HopeNet network. However, based on the prediction results from HopeNet and FSANet, these methods struggle in scenarios involving changes in illumination and self-occlusion. The accessibility of sensors and the development of RGB-D cameras at an increasingly affordable rate have led to the integration of red, green, blue, and depth (RGB-D) images into the head pose estimation process, significantly enhancing accuracy. A head pose estimation method based on Kalman filtering and random forest regression was proposed [20]. This method utilizes depth information from a depth image to estimate head pose. Compared to RGB images, depth images contain more information, thus enhancing head pose estimation accuracy. Wang et al. [21] obtained promising results in head pose estimation by employing point cloud fusion and registration methods, further optimizing the process with a particle filter. Shihua et al. [22] utilized RGB images to convert depth maps into point clouds and proposed a new deep learning network called head pose estimation network (HPENet). This network accepts point cloud data as input and employs HPENet for feature extraction; however, this method exhibits significant estimation errors for extreme head pose angles. In [23], PointNet++ combined with graph CNNs (GCNNs) was employed as a feature extraction network to transform head pose estimation from a regression problem to a classification problem, addressing the issue of large estimation errors in significant head pose angles.

Many existing head pose estimation methods rely on a singular data source, typically utilizing either an RGB or depth image as the input to establish the mapping relationship between 2D and 3D spaces. However, these methods often fall short of delivering accurate or efficient predictions. Consequently, integrating multiple data sources with complementary characteristics becomes imperative for enhancing the overall perception capabilities. This study employed depth and RGB images to generate point cloud data representing the human head. Introducing spatial self-attention mechanisms into PointNet involved replacing the multilayer perceptrons (MLPs) within PointNet with feedforward residual MLPs, resulting in an enhanced PointNet network for extracting spatial features from the point cloud data. Additionally, the ResNet network concurrently extracted textural features. Subsequently, the postprocessing stage fused the features extracted from both networks. Each subnetwork responsible for head pose estimation included fully connected and SoftMax layers, sharing the combined features derived from both point cloud and image data. Moreover, Gumble functions were used to probabilistically encode head pose labels, while the network updated its parameters through a combination of Kullback–Leibler (KL) divergence and expected regression loss. To validate the method’s reliability, the BIWI depth and RGB images were utilized as training and testing datasets for the model. The experiments demonstrated that the proposed method achieved a high accuracy in estimating head poses, particularly when evaluated on the publicly available BIWI dataset.

The remainder of this paper follows the structure below. Section 2 introduces discretization methods and compares them with a Gaussian discretization method. Section 3 outlines the model, categorizing it into four parts and providing separate introductions for the feature extraction, feature fusion, feature scoring, and prediction modules. Section 4 introduces and deliberates on the dataset utilized for the experiments, followed by an in-depth exploration of the conducted experiments and their results. Finally, Section 5 offers a summary of the entire paper and presents conclusions derived from the discussed findings and results throughout the document.

## 2. Discretization of Head Pose Labels

Currently, two primary regression approaches exist. The first is direct regression, which may not accurately predict samples affected by noise. The second method involves regression with classification constraints. In the realm of image-based head pose recognition, Ruiz et al. employed a classification layer to comprehend the data distribution and discretize the regression space. They transformed the direct regression of head pose angles into a data distribution process, generating expected values within predefined classification intervals. The process of computing head pose angles included assigning weights to the predicted values based on their corresponding angles and calculating the regression loss. Subsequently, a combination of classification and regression weights was applied to adjust the contribution of different data distributions during network training. However, head pose angles are arbitrary and continuous within specific ranges. A classification-based approach for head pose estimation might introduce more significant errors, particularly when the input samples exhibit high correlations and low discriminative power. The application of a classification method may result in placing two closely related samples into the same class. To mitigate such issues, a study [24] introduced a label-wise distribution learning approach. This approach transforms a set of Euler angles x={y,p,r} for head poses into discrete Gaussian distribution labels. Here, “x={y,p,r}” denote the yaw angle, pitch angle, and roll angle of the head’s orientation, respectively. These Gaussian labels offer more comprehensive information compared to the original head pose labels, as they account for the actual scalar labels and relationships between neighboring elements within the accurate labels. However, the feature symmetry of Gaussian functions might not adequately capture visual differences in head pose images. To address this, a novel method for discretizing head pose labels was introduced in this study. This method involves both Gaussian and Gumbel functions [25], as illustrated in Equation (1), where β, $s_{l}$, δ, and y represent the scale parameters, position parameters, variance of Gaussian functions, and head pose label values, respectively.

(1) $$\left\{\begin{array}{l} \frac{1}{\beta }\times exp(exp(\frac{-\left(y-s_{l}\right)}{\beta })-\frac{\left(y-s_{l}\right)}{\beta })\;\beta =3,\;y>0\\ exp(\frac{-(y-s_{l}{)}^{2}}{2\delta^{2}})\;\;\delta =1,\;y=0\\ \frac{1}{\beta }\times exp(exp(\frac{-\left(y-s_{l}\right)}{\beta })-\frac{\left(y-s_{l}\right)}{\beta })\;\beta =3,\;y<0 \end{array}\right.$$

**Figure 1:**
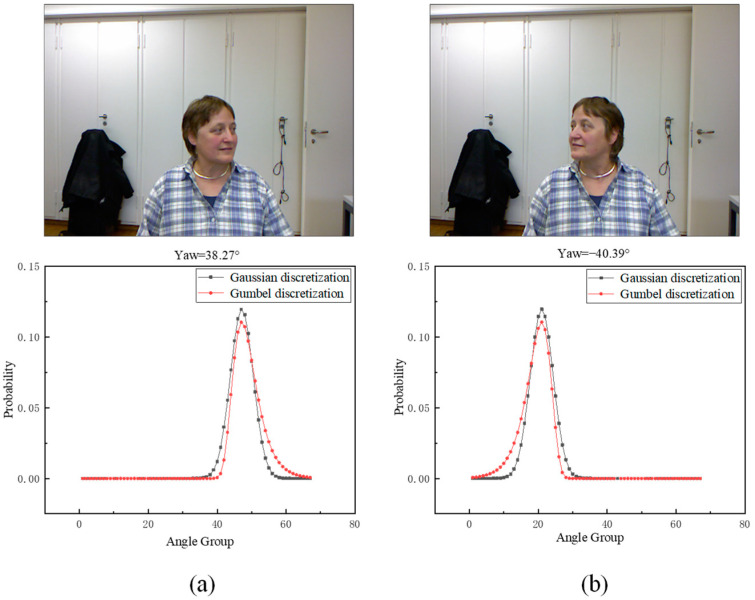
The top is the RGB image, and the bottom is the head pose label corresponding to the RGB image. (**a**) When the yaw angle is 38.27°, the discretized results of the head pose. (**b**) When the yaw angle is −40.39°, the discretized results of the head pose.

The discrete head pose label distributions for angles of 38.27° and −40.39° are illustrated in Figure 1, where the angle interval is 3°, and the values of δ and β are one and three, respectively.

As shown in Figure 1, the range of [−100, 100] is partitioned into 66 intervals with a spacing of 3, and the values of the current head pose are computed separately for each of these 66 intervals. In Figure 1a,b, it becomes evident that Gumbel-based discrete labels exhibit more logical consistency compared to Gaussian-based labels. For instance, visually, 38.27° and −40.39° align with a rightward and leftward tilt, respectively, aligning with the non-feature characteristics of the Gumbel distribution. This alignment reflects the intuitive angular intensity of the head pose in a visual context.

There exist various methods to describe the similarity between the two distributions. Following the network’s output through the SoftMax layer, this study utilized the KL divergence to quantify the similarity between the accurately observed and predicted label distributions, as shown in Equation (2).

(2) $$T=-\sum_{k}y_{k}\ln{\hat{y}}_{k}$$

where $y_{k}$, ${\hat{y}}_{k}$, and k represent the accurate discrete distribution labels, the distribution predicted by the network, and the number of classification intervals, respectively.

Inspired by Ruiz et al., alongside measuring the distinction between label distributions, we implemented the expected regression to quantify the disparity between the actual and estimated head poses. This procedure involved computing the absolute difference between the product of the maximum component of the angular probability distribution and the corresponding angular interval representative value subtracted from the correct head pose angle, as depicted in Equation (3).

(3) $$H=\left|\sum_{n=1}^{k}p_{k}\times {\hat{y}}_{k}-y\right|$$

where y is the true head pose angle; ${\hat{y}}_{k}$ is the distribution predicted by the network, and $p_{k}$ is the representative value of the angle interval corresponding to each category in the predicted label distribution. Finally, the proposed head pose estimation loss function is defined as follows:

(4) L=T+α×H

where α is a weighting parameter that balances the two components of the loss function.

## 3. Head Pose Estimation Network

**Figure 2:**
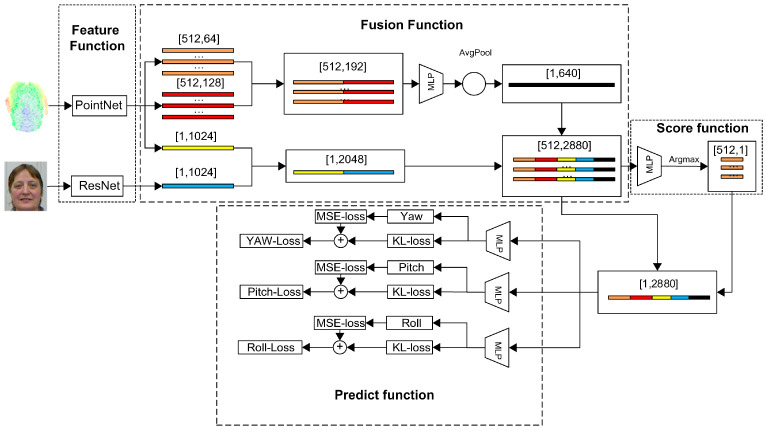
Head pose estimation network. Different colors represent the characteristics of different stages. The network is mainly divided into four modules: feature function, fusion function, score function, and predict function.

The head pose estimation network proposed in this study comprises four principal components, as depicted in Figure 2. The algorithm employs a feature function to extract features from both image and point cloud data. Moreover, a fusion function is utilized to combine the extracted features obtained from the point cloud and image data. Additionally, the algorithm incorporates a score function responsible for assigning scores to each point cloud feature, ultimately selecting the feature with the highest probability value as the final feature. Finally, the algorithm uses a prediction function to estimate the head pose by considering the feature with the highest score.

### 3.1. Feature Function Module

**Figure 3:**
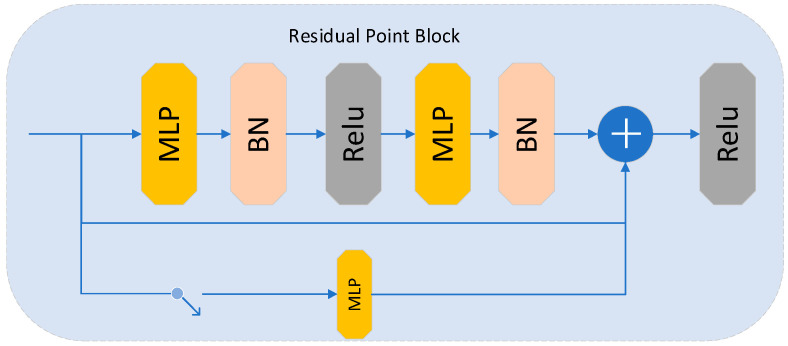
Feedforward residual MLP module.

While neural networks have proven effective in fitting nonlinear functions, this study identified a limitation in capturing high-frequency information within the point cloud when directly inputting the point cloud coordinates into the network for the head pose estimation task. As reported in [26], employing positional encoding functions to project inputs into a higher-dimensional space before feeding them into the network can enhance its capability to learn high-frequency features. Therefore, this study implemented the frequency-encoding method proposed in [27] to transform the coordinates of the point cloud input into a higher-dimensional space. Encoding Equation (5) is expressed as follows:

(5) $$p(x)=(\sin(2^{0}\pi x),\cos(2^{0}\pi x),....,\sin(2^{L-1}\pi x),\cos(2^{L-1}\pi x))$$

where *x* presents a point within the point cloud coordinates, and $L\in N^{*}$ indicates the dimension of the positional encoding. The PointNet network, a deep learning architecture introduced by Charles et al. [28], is specifically designed to process unstructured point cloud data directly. This network incorporates a spatial transformer network comprising a multilayer perceptron to address issues related to translation and rotational invariance within the point clouds. In the model presented here, we incorporated the approach outlined in [29], replacing the MLP structure in the PointNet network with a feedforward residual MLP network to alleviate issues related to gradient explosion and vanishing caused by the depth of the network. Refer to Figure 3 for an illustration of the model’s structure.

Two MLPs primarily constitute this module, with the output of the last MLP layer added to the input data. This mechanism effectively addresses issues related to gradient explosion and gradients resulting from network depth. The core process is described by Equation (6).

(6) $$\left\{\begin{array}{l} y=F(x,w_{i})+x\\ y=F(x,w_{i})+wx \end{array}\right.$$

where $F(x,w_{i})$ represents the residual module to be learned, and $w_{i}$ and w represent the parameters for various MLP layers within the residual structure, encompassing nonlinear and linear transformations, respectively. These parameters correspond to the parameters for the linear mapping MLP, ensuring consistency in the input and output sizes in cases where the dimensions of the input data and MLP output differ.

**Figure 4:**
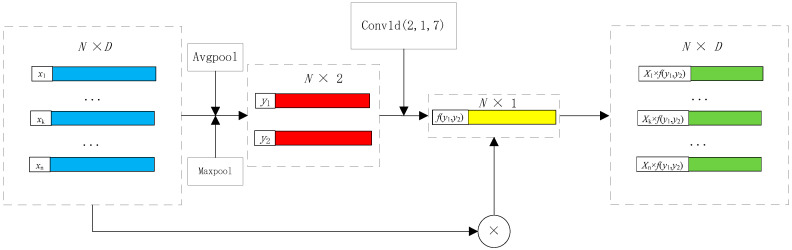
Spatial self-attention module.

In the conversion of depth maps into point cloud data, noise in the depth images often generates outliers and noisy points in the resulting point cloud. To tackle this issue, a spatial self-attention mechanism is integrated into PointNet to calculate the weight of each point. This integration reduces the influence of outliers and noisy point features within the network, subsequently enhancing the accuracy of head pose estimation. Figure 4 provides an illustration of the self-attention mechanism.

**Figure 5:**
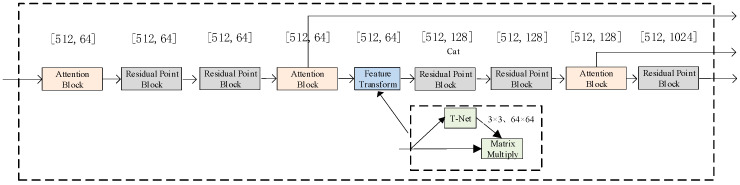
Feature extraction module. The network consists of five residual point blocks, three attention blocks, and a feature transform.

In Figure 4, N represents the number of point clouds, and D denotes the features for each point; the algorithm acquires the maximum and average values along the channels at each point. These two outcomes are then stacked together. A one-dimensional convolution is employed to align the number of channels to one. By utilizing a sigmoid function, the weights for each point are constrained within the range [0, 1]. Finally, the weight values are multiplied by the original values. Image features are extracted using a ResNet network. Figure 5 illustrates the comprehensive structure of the system.

### 3.2. Fusion Function Module

In [30], a method was proposed where features extracted through a traditional feature extractor (Gabor Filter) and those directly extracted using CNNs are fused in later stages. This approach effectively captures low-frequency and high-frequency information from data, leading to promising results in gesture recognition tasks. Additionally, in [31], the authors proposed a multi-level fusion module that integrates high-, medium-, and low-level features from RGB and thermal infrared images. This module effectively aggregates features, providing enhanced semantic information for urban scene segmentation. The aforementioned study highlights the efficacy of sensible feature fusion in efficiently aggregating features, enabling the network to acquire more information and, thus, enhancing the robustness of the model. Taking inspiration from [32,33,34], the point cloud features $y_{1}^{point}$ and $y_{2}^{point}y_{3}^{point}$ are concatenated. Subsequently, this combined feature is mapped to a higher-dimensional space through fully connected layers and is globally averaged to obtain a new global feature $y_{4}^{point}$. Simultaneously, the global features of the point cloud $y_{3}^{point}$ and $y_{4}^{point}$, the global features of the image $y_{1}^{RGB}$, and the features of each point in the point cloud $y_{1}^{point}$ and $y_{2}^{point}y_{3}^{point}$ are concatenated to derive the final fused feature. This approach facilitates a comprehensive fusion of point clouds and image features, as the feature encompassed the global characteristics from each stage of the image and point cloud, along with the individual characteristics of each point in the point cloud.

### 3.3. Score and Prediction Function Module

The fused feature $y_{1}^{funsion}$ is processed through an outer fully connected layer and is then subjected to a SoftMax operation to derive the scores for each point.

**Figure 6:**
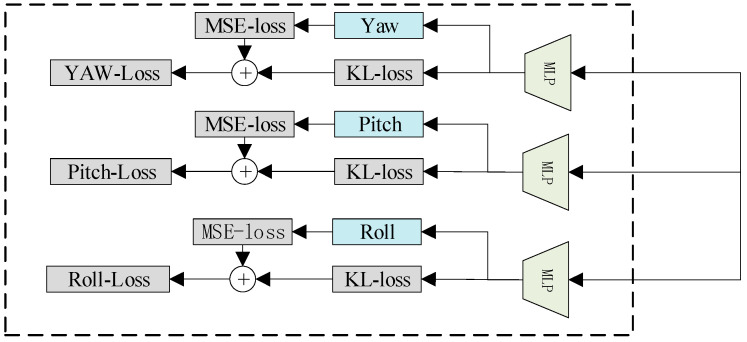
Classification and regression module.

The prediction module is primarily composed of a classification module and a regression module, as illustrated in Figure 6. The classification module comprises three distinct MLPs, each responsible for estimating different poses of the human head. These MLPs function independently, with the parameter “λ” determining the number of neurons in each MLP. On the other hand, the regression module computes the final head pose prediction by summing the product of the representative values for each classification interval based on the classification results.

The prediction module initially learns data distribution by utilizing a classification layer and discretizing the regression space. Head pose angles are then directly converted into data distribution and anticipated values within predetermined classification intervals. These predicted values undergo weighting with corresponding angles to compute the head pose angles for regression loss. Subsequently, the contribution of various data distribution values to network training are adjusted by employing a weighted combination of classification and regression. Following the output production by the SoftMax layer, KL divergence is used to assess the similarity between the true and predicted label distributions. Additionally, to quantify disparities between the actual and estimated head poses, we employed expected regression. The proposed head pose estimation loss function is defined as follows:

(7) $$L=-\sum_{k}y_{k}ln{\hat{y}}_{k}+\alpha \times \left|\sum_{n=1}^{k}p_{k}\times {\hat{y}}_{k}-y\right|$$

where y represents the true head pose angle values; ${\hat{y}}_{k}$ represents the distribution predicted by the network; $p_{k}$ denotes the representative values of the angle intervals corresponding to each category in the network’s predicted distribution labels, and α serves as an essential weight parameter that balances the two components of the loss function.

## 4. Experimental Results

### 4.1. Data Source

This study utilized the BIWI dataset [35], which includes over 15,000 RGB-D images at a resolution of 640 × 480. The dataset contained information from more than twenty individuals (six females and fourteen males) with head yaw angles starting from ±75° and pitch angles from ±60°. Each frame within the dataset consists of an RGB image, a depth image, corresponding ground-truth head pose parameters, and three-dimensional positional parameters for the human head center. The BIWI dataset is commonly used for training 3D head pose models.

### 4.2. Experimental Parameters

The experimental setup comprised an 11th Gen Intel(R) Core(TM) i5-1135G7 2.40 GHz processor, 16.0 GB of system RAM, and a 64-bit Windows 10 operating system. It also included a GTX 3060 GPU with 12 GB of VRAM, and the development environment was based on Python 3.7, using the Torch 1.71 framework. The initial step in this study involved using a face detector to identify facial regions, followed by cropping the image based on the detected face’s position. Subsequently, the network employed the cropped RGB head image and the depth image to convert the human head into point cloud data and extract point cloud features. The interval angle λ was initially set to 1° when creating head pose labels, resulting in 200 classes with β set to 3. Discrete distribution labels were computed for each image in the BIWI dataset. The loss function, which combined classification and regression components, was used to update network parameters via backpropagation. The weight coefficient α in the loss function was initially set to 0.5. Experiments were conducted using the first sixteen datasets from the BIWI dataset as the training set, with the remaining eight datasets utilized as test set α. Cross-validation was performed using sets 1, 7, 13, and 19 as individual test sets to underscore the reliability of the proposed method. Additionally, for a comparative analysis with the prevalent head pose estimation methods, datasets 11 and 12 were used as the test sets, while the remaining data served as the training set. Notably, no data augmentation was applied in these experiments owing to the nature of the point cloud data generated from the depth images, representing only the surface of the human body, which would alter with image rotation. The selected hyperparameters included a batch size of 16, an initial learning rate of 0.001, and a decay in the learning rate of 0.7 every 40 epochs. The training process spanned 100 epochs, using the mean absolute error defined in Equation (8) as the evaluation metric:

(8) $$MAE=\frac{1}{N}\sum_{n=1}^{N}\left\|Y_{predict}-Y_{real}\right\|$$

where *N* represents the total number of frames; $Y_{predict}$ represents the head pose estimated by the model, and $Y_{real}$ represents the true head pose angles.

### 4.3. Experimental Results

#### 4.3.1. Data Processing Result

**Figure 7:**
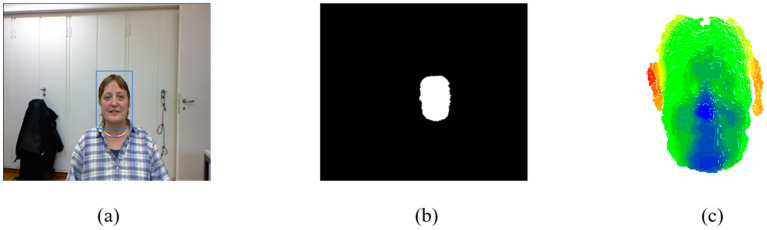
Data source. (**a**) RGB image. The head position in the RGB image. (**b**) Head mask. In the head mask, the white region is the head, while the black region is the background. (**c**) Point cloud. From the depth map to the point cloud.

We utilized the head mask and head center position information from the BIWI dataset to derive candidate boxes for the head, thereby generating point cloud data, as depicted in Figure 7.

**Figure 8:**
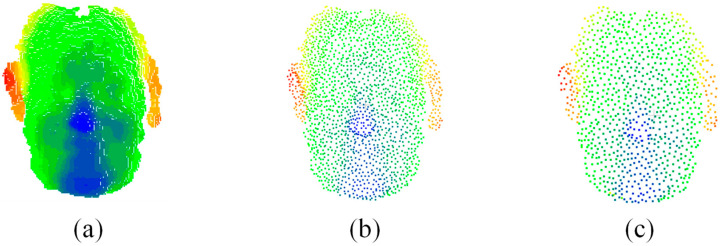
Point clouds at different scales. (**a**) Primary point cloud. (**b**) Downsampling the original point cloud to 1024. (**c**) Downsampling the original point cloud to 512.

The volume of point cloud data significantly impacts the accuracy and speed of head pose estimation. Point clouds containing an excessively large quantity of data often encompass redundant information. Directly inputting such voluminous data into the network can lead to prolonged computation times. Conversely, having too few points in the point cloud may result in the loss of local feature information. To strike a balance between accuracy and enhanced model inference speed, this study uniformly downsampled the point cloud data to 512 points, as demonstrated in Figure 8.

#### 4.3.2. Ablation Experiment Results

This study explored the values of α and conducted experiments with α sets of 0.1, 1, and 2. The initial 16 sets from the BIWI dataset constituted the training set, while the remaining sets served as the test sets for the experiments. Table 1 showcases the experimental results.

The α value represents the weight of the expected regression loss within the overall loss function. When this weight is low, the network tends to focus more on retaining the distribution of discrete data, potentially affecting accurate predictions. Conversely, when the weight is high, the network may learn the data themselves but could fail to capture the relationships between adjacent elements of the true labels α. Setting α to 0.1 resulted in the model exhibiting its poorest performance on the test set, as indicated in Table 1. In contrast, setting α to 1 led to the highest accuracy achieved. Therefore, for the subsequent experiments in this study, α was set to 1. Regarding the self-attention mechanisms, a comparison was drawn between training a standalone PointNet network and a PointNet network. The results of this comparison are presented in Table 2.

**Figure 9:**
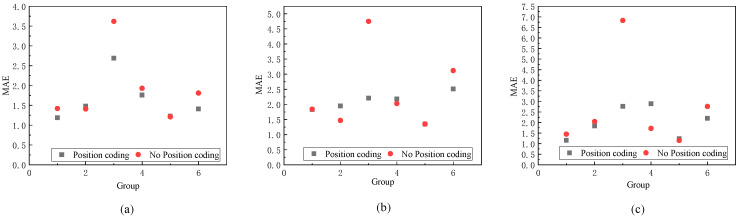
When comparing model prediction accuracy, within the same dataset, between instances with and without positional encoding. (**a**) The predictive accuracy of the model on yaw angles. (**b**) The predictive accuracy of the model on pitch angles. (**c**) The predictive accuracy of the model on roll angles.

In the table, “S-A” denotes the incorporation of spatial attention mechanisms, while “R” and “P” represent the data sources as images and point clouds, respectively. The results indicate that integrating self-attention mechanisms into PointNet enhanced the accuracy of head pose estimation. Moreover, the fusion of point cloud features with image features for head pose estimation yielded a higher accuracy compared to estimating the head pose from a single data source. Additionally, encoding the position of the network input enabled the model to capture more high-frequency information, thereby enhancing the accuracy of head pose estimation. To verify the effectiveness of this approach, comparative experiments were conducted, using the 1st, 3rd, 6th, 8th, 17th, and 23rd groups as test sets. Simultaneously, the remaining data comprised the training set. Figure 9 presents the experimental results.

Evidently, head pose estimation utilizing positional encoding in the model’s input showcased more accurate results compared to head pose estimation without positional encoding. The most notable enhancement was observed when employing the 6th group of data as the test set, resulting in an accuracy increase of approximately 3° for both the roll and yaw angles.

#### 4.3.3. Model Evaluation and Comparison

To authenticate the reliability of the proposed method, it was juxtaposed with other mainstream methods for head pose estimation. The experiments utilized the 11th and 12th groups as test sets, while the remaining groups constituted the training sets. Table 3 depicts the experimental results.

**Figure 10:**
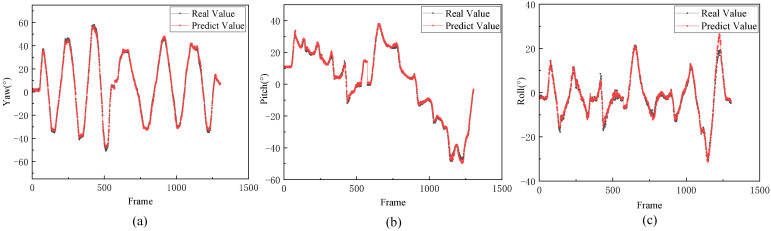
When using the 11th and 12th instances as the dataset, a comparison between the model’s predicted values and the ground truth. (**a**) Comparison on yaw angles. (**b**) Comparison on pitch angles. (**c**) Comparison on roll angles.

As observed, the accuracy of head pose estimation using depth images surpassed that of RGB images due to the richer information present in-depth images. Moreover, head pose estimation utilizing point cloud data achieved even higher accuracy than the preceding methods. This is primarily attributed to the more pronounced spatial information within the point cloud data compared to the depth images. Among the nine head pose estimation methods scrutinized, the approach proposed in this study yielded the most accurate estimation results. Figure 10 juxtaposes the ground-truth values with the predicted values of the yaw, roll, and pitch angles within the test set. Notably, when handling both small and large pose angles, the head poses estimated by the method outlined in this study closely corresponded to the ground-truth values. Using the Gumbel distribution instead of the Gaussian distribution led to an increase of 0.27° in the pitch angle and 0.06° in the roll angle.

**Figure 11:**
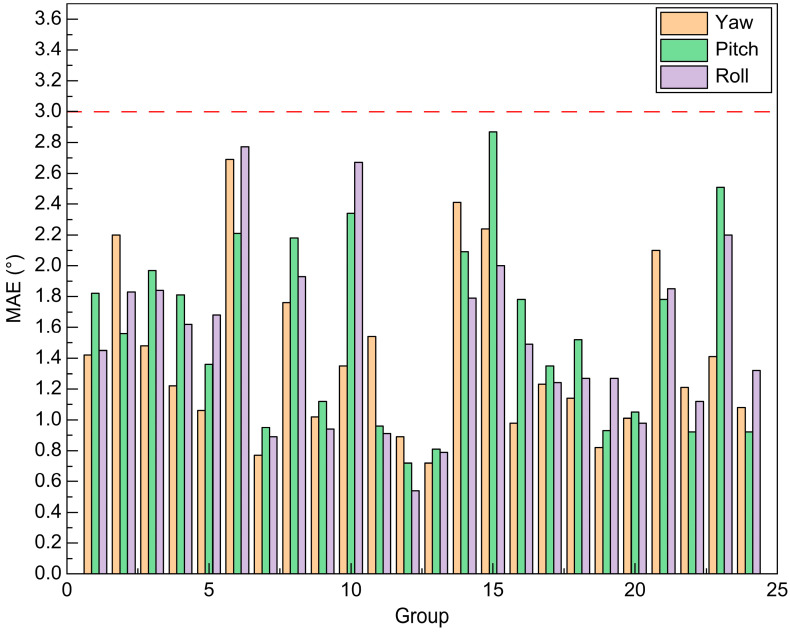
When each set of data in the dataset is used as a test set, the model’s predicted results are compared to the ground truth, and the average absolute error is calculated. The red dotted line represents the upper limit of the model’s prediction accuracy.

To evaluate the model’s generalizability, each of the 24 data groups was individually used as a test set, while the remaining groups served for training, as illustrated in Figure 11. Across the 24 test experiments, it became apparent that the average absolute errors for each angle were all below 3°. Notably, the 7th, 11th, 12th, and 13th groups showcased average fundamental errors of less than 1° for each angle. These experimental outcomes strongly suggested that the proposed method surpassed the BIWI head pose recognition accuracy.

**Figure 12:**
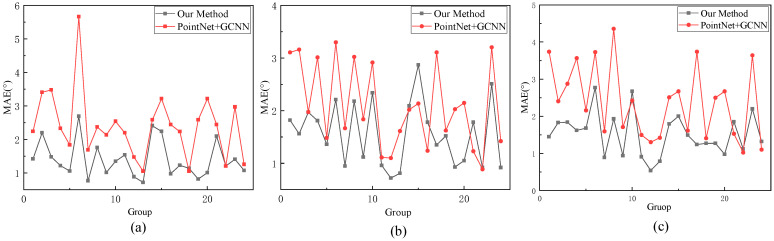
Comparison of different methods on the BIWI dataset. (**a**) The comparison of different methods in terms of yaw angle prediction accuracy. (**b**) The comparison of different methods in terms of pitch angle prediction accuracy. (**c**) The comparison of different methods in terms of roll angle prediction accuracy.

In [18], the authors attained the most favorable head pose estimation outcomes by integrating PointNet++ and GCNN. For the comparative analysis, cross-validation was conducted, designating each dataset as a test set. In this study, similar cross-validation techniques were employed, using each dataset as a test set, and the obtained experimental outcomes were compared with those detailed in [23]. The comparative results are illustrated in Figure 12.

For head pose estimation, point cloud data contain more information than RGB data, while the global information extracted from image features can complement the point cloud information. Experimental results also confirmed that the method presented in this study consistently achieved higher head pose estimation accuracy than the approach described in [23], with errors for head pose angles in groups 1, 13, and 19 all being less than 1°.

**Figure 13:**
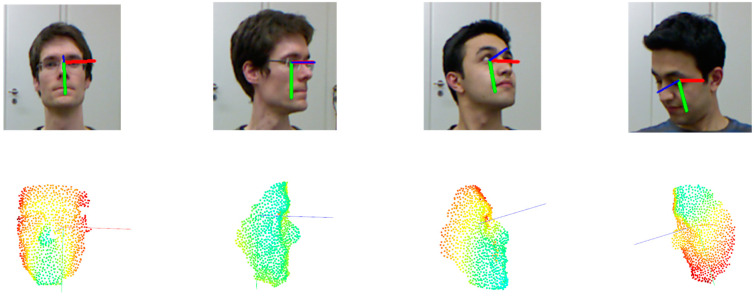
Visualization of partial test set results. The blue, green, and red colors respectively represent yaw, pitch, and roll angles. The top and bottom sections show the RGB and point cloud visualizations, respectively.

This study provides visual results of the head pose estimation for small and large pose angles in the 11th and 12th groups to demonstrate the approach’s advantages more intuitively. Figure 13 shows that the proposed method can provide accurate predictions even in significant facial occlusion conditions and large pose angles. This reflects the robustness of the approach for facial occlusion conditions and large head pose angles.

## 5. Discussion

### 5.1. Comparison with Existing Methods

In the above experimental results, this paper compared its method with those from references [15,16,23,36,37,38,39] on the BIWI dataset, and the experimental results are shown in Table 3 From the results, it can be observed that the model proposed in this paper achieved a higher accuracy. From Table 3, it is evident that the accuracy of estimating head pose solely from RGB images is lower compared to the accuracy obtained from depth images. This is because depth images contain more information compared to RGB images. Furthermore, head pose estimation using point cloud data achieved an even higher accuracy than the previous two methods. This is primarily because of the more pronounced spatial information in the point cloud data than in the depth images.

Additionally, this paper conducted cross-validation on the BIWI dataset and independently compared the proposed method with the model in reference [23]. Experimental results demonstrate that the approach presented in this paper performs optimally across all test sets. Moreover, even in cases where large pose angles lead to the absence of head point clouds, the proposed method effectively estimates head pose. This suggests that image features can serve as a complement to missing point cloud information. Furthermore, favorable head pose estimation results can be achieved through a judicious feature fusion method.

### 5.2. Future Research

The point cloud and image features extracted by different networks are distinct. Rich feature information is beneficial for head pose estimation accuracy; however, redundant features can decrease the inference speed of the model. This study utilized improved PointNet networks for point-cloud feature extraction and employed ResNet networks to extract image features. This paper did not introduce any improvements in the image feature extraction network. In feature fusion, the consideration of time complexity was also omitted. Using image features as a supplement to the missing information in the point cloud can enhance the accuracy of head pose estimation. Still, it comes at the cost of increased model inference time.

In the future, researchers should explore models more suitable to extracting features from point clouds and images. Additionally, concerning the fusion of these features, consideration will be given to time complexity and feature redundancy. One approach involves early mapping of the point cloud onto the image, followed by extracting features from the corresponding pixel positions of the point cloud in later stages for fusion.

## 6. Conclusions

We generated head point clouds from depth maps and discretized head pose labels using the Gumbel function. The MLP in PointNet was substituted with a residual MLP module, incorporating a self-attention mechanism to extract point cloud features. Additionally, we utilized a ResNet network to extract global features from images. The features from both sources were then fused, and the SoftMax function was applied to compute scores for each point, identifying the features of the point with the highest score. During prediction, the model input the highest score features into a classification block and estimated the final pose through a fully connected layer using a softmax function. The experimental validation of the proposed method on the BIWI dataset resulted in errors of 1.18°, 0.67°, and 0.68° for the yaw, pitch, and roll angles, respectively. Compared to widely used head pose estimation methods, the proposed method demonstrated a significantly improved accuracy in head pose estimation.

## Figures and Tables

**Table 1:** Influence of α on head pose estimation.

α	Yaw (°)	Pitch (°)	Roll (°)	Mean (°)
0.10	1.67	1.85	2.26	1.92
1.00	1.38	1.34	1.76	1.49
2.00	1.36	1.43	1.85	1.54

**Table 2:** Comparison of head pose estimations obtained for different data sources and methods.

Method	Yaw (°)	Pitch (°)	Roll (°)	Mean (°)
S-A + P + R	1.38	1.34	1.76	1.49
S-A + P	1.62	2.05	2.07	1.91
P	1.44	2.59	2.17	2.06

**Table 3:** Comparison between different head pose estimation methods. PGCNN stands for point graph convolutional neural networks.

	Data	Yaw (°)	Pitch (°)	Roll (°)	Mean (°)
FineGrained [15]	RGB	3.29	3.39	3.30	3.23
FSANet [16]	RGB	4.96	4.27	2.76	4.00
PGCNN [23]	Point	1.82	1.09	1.39	1.42
Multi-task [36]	RGB	4.30	3.60	3.40	3.76
CoarseFine [37]	RGB	4.76	5.48	4.29	4.84
POSEidon [38]	Depth	1.70	1.60	1.80	1.70
RobustMode [39]	Depth	2.40	2.20	2.10	2.10
Our (Gauss)	RGB + Point	1.12	0.94	0.74	0.93
Our (Gumbel)	RGB + Point	1.18	0.67	0.68	0.84

## Data Availability

The data presented in this study are openly available in Random Forests for Real Time Head Pose Estimation (ethz.ch).
